# How necessary it is to perform a ventilation scan in patients with a history of COVID-19 to rule out pulmonary thromboembolism?

**DOI:** 10.22038/aojnmb.2024.77934.1550

**Published:** 2025

**Authors:** Farivash Karamian, Roham Nikkhah, Mohammad Ghorbani, Elham Rahmanipour, Mohammad Mohammadi, Emran Askari, Ramin Sadeghi

**Affiliations:** 1 Nuclear Medicine Research Center, Mashhad University of Medical Sciences, Mashhad, Iran; 2 Isfahan Medical Students Research Committee (MSRC), Isfahan University of Medical Sciences, Isfahan, Iran; 3 Orthopedic Research Center, Faculty of Medicine, Mashhad University of Medical Sciences, Mashhad, Iran; 4 Immunology Research Center, Faculty of Medicine, Mashhad University of Medical Sciences, Mashhad, Iran; 5 School of Medicine, Iran University of Medical Sciences, Tehran, Iran

**Keywords:** SPECT/CT scan, COVID-19, Pulmonary embolism, Ventilation scan

## Abstract

**Objective(s):**

This study evaluated the necessity of a ventilation scan in patients suspected of PE with a history of COVID-19 infection.

**Methods:**

This was a cross-sectional study of patients with PCR-confirmed COVID-19 and suspected PE at a tertiary care hospital in 2020. They underwent ventilation/perfusion (V/Q) scintigraphy using single-photon emission computed tomography/computed tomography (SPECT/CT) and CT scans with or without contrast. Two blinded nuclear medicine physicians interpreted the images for PE and COVID-19. Clinical and laboratory data were extracted and analyzed.

**Results:**

96 patients with suspected PE and COVID-19 infection. The study excluded eight patients who could not undergo ventilation scans and confirmed PE in five patients with multiple mismatched V/Q defects on SPECT/CT. The study ruled out PE in 83 patients who had either regular perfusion scans, perfusion defects with COVID-19 features, or matched V/Q defects. The study found that the prevalence of PE was 5.68%, and the necessity of ventilation scans was 28.40% in this population.

**Conclusion:**

It was found that PE was present in 5.68% of the patients, and ventilation scans were needed for 28.40% of the patients to confirm or exclude it.

## Introduction

Although COVID-19 was primarily considered a respiratory illness, rapidly accumulating data suggest that COVID-19 is associated with a high incidence of venous thromboembolic complications (1).

 Computed tomography of pulmonary arteries (CTPA) and V/Q scan are the two most common and widely practiced testing modalities to diagnose PE (2).

 CTPA is capable of direct visualization of PE. However, the suitability of CTPA as a primary diagnostic modality is questionable because of the radiation exposure, specific contraindications, and a significant percentage of false positive results (3, 4).

 V/Q scintigraphy has been an authentic diagnostic method for diagnosing PE; therefore, many patients were referred for V/Q scans for suspected PE during the COVID-19 pandemic (2, 5-7).

 However, the biggest concern in the COVID-19 era was that the ventilation procedures increased the potential risk of contamination by aerosol secretion and expired air.

 During the pandemic, the nuclear medicine

community has tried to implement approaches to performing lung scintigraphy that do not compromise the safety of staff and patients (8).

 The Society of Nuclear Medicine and Molecular Imaging (SNMMI) responded promptly to the fast spread of the pandemic by releasing a statement on March 19, 2020, stating that ventilation scans for PE should be performed only when necessary (2021). 

 Although the perfusion-only SPECT/CT strategy is less effective as compared to the standard V/Q imaging, using CT images can show the hypo-ventilated areas of the lung to some extent (9, 10); however, several groups have warned of the possibility of false positive findings in patients with prior history of COVID-19, if the images are interpreted using the perfusion-only strategy (11). These studies favour the ventilate-all strategy instead of the previously mentioned guideline recommendations. 

Finally, the intermediate approach would be a perfusion SPECT or SPECT/CT scan followed by a ventilation scan only when necessary (7, 11-13).

 We evaluated this approach's feasibility and aimed to assess the necessity of a ventilation scan in patients suspected of PE with a history of COVID-19 infection. The secondary aim was to describe practices and imaging findings in this population.

## Methods

***Patient population***

 This retrospective study consists of 88 patients with PCR-confirmed COVID-19 infection and underwent V/Q scintigraphy for suspected PTE from January 2020 onwards. The data was collected on their clinical and laboratory features, such as pulmonary thromboendarterectomy (PTE) risk factors, electrocardiogram (ECG) findings, echocardio-graphic measurements, SpO_2_, and D-dimer levels. A history of lung diseases, such as asthma and chronic obstructive pulmonary disease (COPD) has been recorded.

***VQ imaging algorithm in the COVID-19 era***

 The Q SPECT/CT (or Q SPECT for pregnant patients) has been performed for all patients and evaluated the scans by two independent nuclear medicine specialists. If no perfusion defects were detected on Q SPECT/CT, PTE was ruled out, and a ventilation study was not conducted.

 If there were perfusion defects on Q SPECT/CT, the CT findings were compared to them. If the defects corresponded to the lung parenchymal changes on CT, the PTE ruled out and did not perform a ventilation study. If there were segmental perfusion defects without matching parenchymal changes on CT, a ventilation study using the SPECT/CT method was conducted.

***The perfusion study protocol***

 During the perfusion trial, a supine patient was injected with 1-3 millicuries of ^99m^Tc MAA (macro aggregated albumin) solution, which included 250,000-750,000 particles. The particles and activity levels were decreased for pregnant patients. Eight planar pictures were acquired using a LEAP collimator, with each projection having 500,000 counts and a matrix size of 256×256. A 360-degree single-photon emission computed tomography (SPECT) picture was acquired using a matrix size of 64×64, a projection duration of 12 seconds, and a step size of 3 degrees. The GE dual-head gamma cameras (model NM 670 Discovery) were utilized for imaging purposes.

***The ventilation study protocol***

 The ventilation study was conducted on a different day within 24 hours of the perfusion study for patients with abnormal perfusion scans. A nebulizer system with 740 to 1110 MBq (20–40 mCi) of ^99m^Tc-DTPA (Diethylenetriamine pentaacetate) (Pars Isotope, Tehran) was used to deliver the radioactive tracer to the patients' lungs. Patients inhaled the tracer for approximately 15 minutes before imaging, and imaging commenced once the tracer's counting rate exceeded four times that of their perfusion scan. The ventilation process itself was completed before the patient entered the camera room for imaging. Planar images were acquired in the same eight projections as the perfusion scans with 500K counts each using a LEAP collimator. SPECT/CT acquisition was also performed with the same settings as the perfusion scan. Special radiation safety and COVID-19-related preventive measures, as described previously, were followed.

***V/Q interpretation***

 The V/Q scans were interpreted according to the European Association of Nuclear Medicine (EANM) criteria while incorporating all relevant clinical and additional imaging data. A scan was considered positive for PTE if there was at least one significant segmental or equivalent moderate or small-sized segmental V/Q mismatched defect. In some cases, mainly for clinically unstable patients, the scans were reported using the perfusion-only criteria (i.e., the PISAPED criteria). Therefore, some patients were considered inconclusive because the ventilation scan was unavailable.

## Results

 We performed perfusion scans on all 96 participants (mean age, 53.85±20.69 years). Of these, 28 subjects (including 19 pregnant women and nine others for whom the CT component was omitted due to logistical issues) underwent Q SPECT. The remaining 68 participants underwent Q SPECT/CT. Eight patients had perfusion defects without any corresponding CT abnormalities, requiring a ventilation scan. However, these patients could not undergo ventilation scans due to their overall health conditions, resulting in their exclusion from our analysis. Table 1 summarizes the demographic information of the included patients.

 The median number of defects per case was 2, with an interquartile range of 1 to 3. Bilateral defects were more prevalent (32.95%, n=29) than unilateral defects (28.40%, n=25). Effusions were present in seven cases (7.96%). 

**Table 1:** Demographic information of patients

**Characteristic**	**Number (Percentage %)**
**Female **	
Pregnant	18 (20.45)
Non-pregnant	42 (47.72)
**Perfusion Defects**	
None	33 (37.5)
One	10 (11.36)
Two	12 (13.63)
Three	6 (8.18)
Four	27 (30.68)
**Underlying Disease**	
Asthma	9 (10.22)
COPD	9 (9.09)
Smoking	17 (19.31)
OCP	4 (4.55)
Immobilization	7 (7.94)
Prior/Current DVT	3 (3.41)
Prior PTE	1 (1.14)
Hemoptysis	5 (5.68)
PE Effusion	7 (7.96)

Abbreviations: COPD: Chronic Obstructive Pulmonary Disease, OCP: Oral Contraceptive Pills, DVT: Deep Vein Thrombosis, PTE: Pulmonary Thromboendarterectomy, PE: Pulmonary Embolism

**Figure 1:**
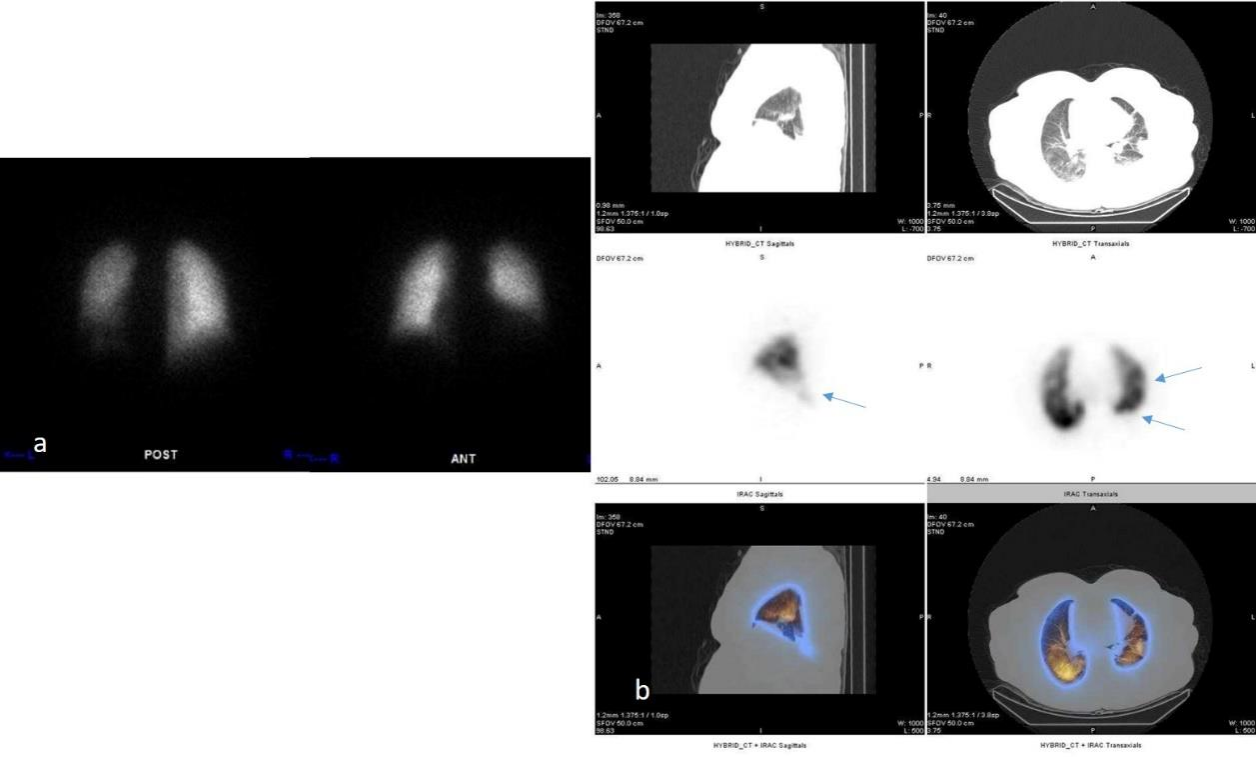
Lung planar (**a**) and SPECT/CT (**b**) perfusion scan of a patient. Perfusion defects in the left lung correspond to CT abnormalities, including consolidations and pleural effusion. The ventilation scan was not done. The scan was not compatible with PE. Blue arrows indicate the observed defects or abnormalities in the images

**Figure 2:**
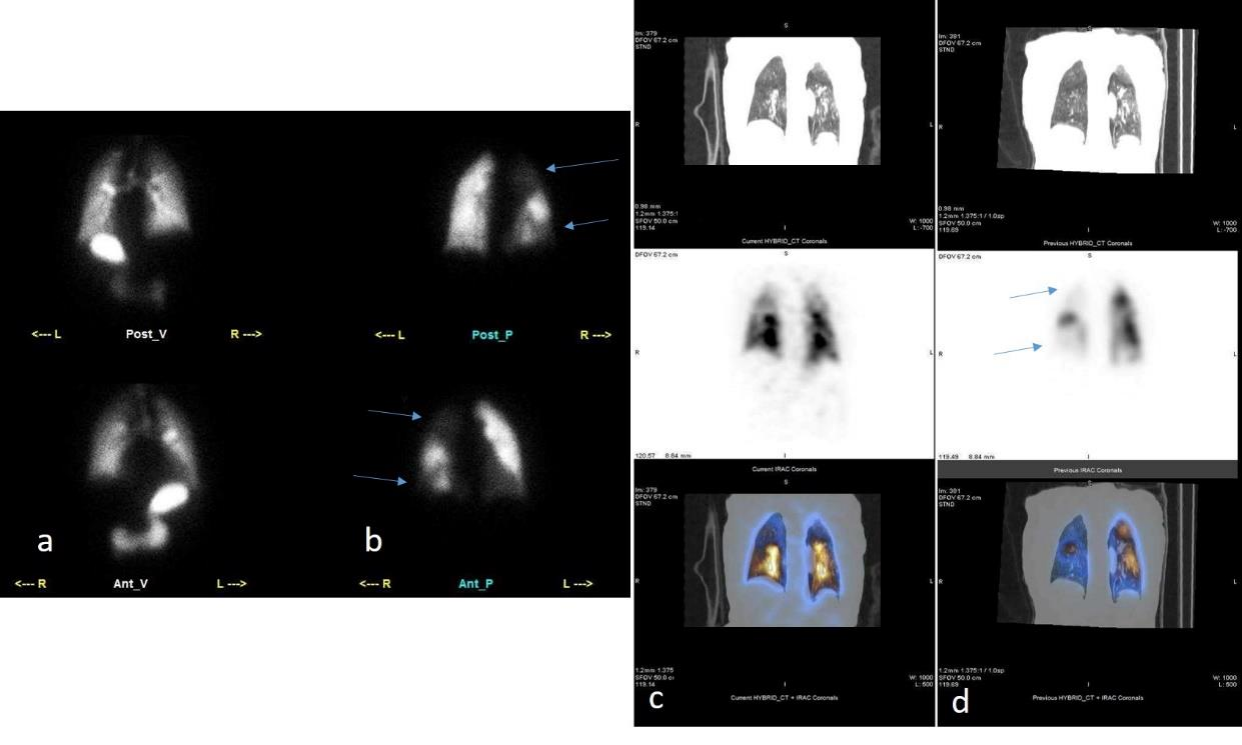
Lung planar V/Q images (**a** and **b**) and SPECT/CT V/Q images **(c** and **d**) of a patient. Multiple mismatched perfusion/ventilation defects are visible in the right lung, which are highly indicative of PE. The blue arrows point to the specific areas where these defects are observed, highlighting the regions of abnormal perfusion that do not correspond with ventilation, characteristic of PE

**Figure 3:**
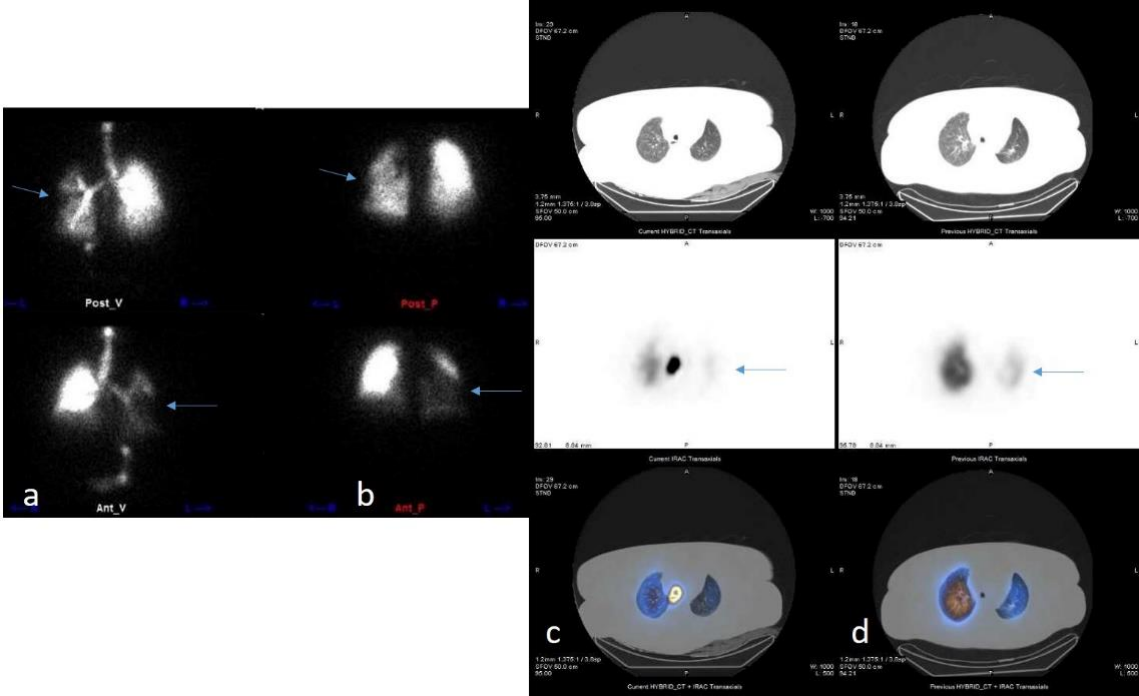
Lung planar V/Q images (**a** and **b**) and SPECT/CT V/Q images (**c** and **d**) of the patient. Matched perfusion/ventilation defects are observed in the left lung without any corresponding CT abnormalities, suggesting that the scan is not compatible with pulmonary embolism (PE). The blue arrows indicate the regions where these matched defects are visible, highlighting areas of concern that correspond to both perfusion and ventilation defects

 Of the 88 patients included in the study, 33 had normal perfusion scans, ruling out the need for a ventilation scan. In 30 patients, perfusion defects matched with CT abnormalities associated with COVID-19 (Figure 1), thus negating the need for ventilation imaging. For25 patients, a ventilation scan was deemed necessary. In five patients, multiple mismatched V/Q defects indicated PE (Figure 2), whereas in 20 patients, matched V/Q defects ruled out PE (Figure 3). 

 The clinical presentations of the 20 patients who exhibited matched V/Q defects without corresponding CT abnormalities were diverse. The most prevalent presenting symptom of these patients was mild to moderate dyspnea. 

 Despite the presence of these symptoms, the CT scans did not reveal any significant findings, thereby eliminating the possibility of consolidations, effusions, or other parenchymal abnormalities that are typically associated with COVID-19 or PE. Most of these patients had a history of chronic respiratory conditions, including COPD and asthma, which may have contributed to the observed V/Q defects. 

 Nevertheless, the absence of CT abnormalities and the matched character of the V/Q defects indicate that these findings were not indicative of acute PE.

 Among the 88 patients in the study, PE was ruled out by perfusion SPECT or SPECT/CT in 63 patients (71.59%) and V/Q SPECT/CT in 20 patients (22.72%). The diagnosis of PE was made in five patients (5.68%). Therefore, in 28.40% of our patients, a ventilation scan was required to confirm or exclude PE. A ventilation scan was deemed necessary for 34.37% of the patients included in the study when we considered the eight excluded patients for whom a ventilation scan could not be performed because of their overall health conditions.

## Discussion

 The use of SPECT and CT is an integral part of the comprehensive management of patients during the COVID-19 pandemic. In a systematic review on the topic of perfusion ventilation scans in COVID-19 patients, an evaluation of 27 relevant articles was conducted (6). They concluded that the combined use of SPECT/CT and lung perfusion scans could alleviate the diagnostic challenges associated with COVID-19. They reported that perfusion-only SPECT/CT could be a helpful tool for ruling out PE in many patients; however, ventilation scans were recommended in some instances, demonstrating nuclear medicine's versatility in managing this complex and multifactorial condition (6, 14, 15). Recent studies have highlighted the critical role of imaging techniques in assessing cardiovascular complications in COVID-19 patients, with myocardial perfusion imaging emerging as a valuable tool for evaluating ischemic heart disease during the pandemic (16).

 Given these complexities, it is essential to evaluate the advantages and disadvantages of different diagnostic approaches. The ventilate-all approach offers comprehensive diagnostic accuracy by identifying ventilation and perfusion defects, reducing false positives and ensuring thorough assessment (17, 18). 

 However, it increases the risk of COVID-19 transmission, requires more time, and is resource-intensive. The intermediate approach, which starts with perfusion imaging and adds ventilation scans only, when necessary, mitigates transmission risks, conserves resources, and is more efficient. However, it may lead to false positives or diagnostic uncertainty, particularly in patients with underlying lung conditions or post-COVID-19 changes, potentially delaying treatment (19). 

 The choice between these approaches should consider clinical context, resource availability, and safety concerns.

 It was initially considered that patients with COVID-19 could undergo lung scintigraphy scans without the ventilation component due to concerns about disease transmission. Several procedures known to generate aerosols, such as ventilation scans, have been identified as possible sources of infection during the SARS-CoV-2 pandemic (14). Consequently, a discussion was held regarding the necessity of including a ventilation examination as part of the V/Q scan process. There was an assumption that omitting the ventilation procedure could reduce the spread of infection. Furthermore, previous studies have indicated that perfusion-only imaging may be able to effectively rule out PE when it yields normal results (20, 21). This approach was influenced by the lack of data regarding the aerosol-generating nature of the procedure and the potential for misdiagnosis of non-thrombotic events (11, 21, 22). A perfusion-only approach has been associated with more false-positive PE diagnoses. In addition to increasing the risks to patients and healthcare workers through closer contact during subsequent diagnostic and therapeutic procedures, it also increases the risk of hemorrhage due to the potential prolonged use of anticoagulants (21, 22).

 Modifications of the conventional workflow framework were proposed to address the issues and balance safety and diagnostic accuracy. At first, a perfusion SPECT could be obtained and be followed by the necessary low-dose CT scan. 

 When structural findings on low-dose CT were insufficient to explain perfusion deficits, a ventilation SPECT could be performed (12). As a result of incorporating a ventilation scan into the diagnostic procedure, various aspects of SARS-CoV-2 can be addressed substantially, thereby enhancing the inclusion of this procedure within the diagnostic framework (12, 14).

 Le Roux and colleagues conducted a critical multicenter study to determine whether ventilation scans are appropriate to rule out PE in patients who have a history of COVID-19 (11). 

 Out of 145 patients, ventilation scans were not required for 82 individuals (57%), as perfusion SPECT or SPECT/CT scans were sufficient. For the remaining 43% (63 patients), a ventilation scan was vital for assessing potential PE. The results of our study paralleled those of Le Roux, with 34.37% of patients requiring a ventilation scan. It was impossible to perform ventilation scans on eight patients due to their general health conditions, highlighting the logistical difficulties encountered during the COVID-19 outbreak. As a result, our findings fit with those of prior studies suggesting a multimodal approach for diagnosing PE in this particular population of patients (11, 12, 14).

 Our findings, however, are subject to several limitations that may affect their reliability and generalizability. Firstly, the small sample size raises questions about the statistical validity of our findings. This limitation and the fact that our data were obtained from a single medical center may limit our ability to generalize our findings to a broader population. We recommend more rigorous study designs, such as prospective cohort studies, involving a larger and more diverse sample size to improve future research's reliability and applicability.

## Conclusion

 In conclusion, this study on 96 participants, primarily focusing on detecting PE, offers significant insights into the diagnostic process during the COVID-19 pandemic. The study successfully ruled out PE in 71.59% of the cases using perfusion only SPECT or SPECT/CT. A ventilation scan was essential for 28.40% of the patients to confirm or rule out PE.

## References

[1] Jayarangaiah A, Kariyanna PT, Chen X, Jayarangaiah A, Kumar A (2020). COVID-19-Associated coagulopathy: an exacerbated immunothrombosis response. Clin Appl Thromb Hemost..

[2] Zuckier LS (2022). Safe pulmonary scintigraphy in the era of COVID-19. Semin Nucl Med.

[3] Bajc M, Schümichen C, Grüning T, Lindqvist A, Le Roux PY, Alatri A (2019). EANM guideline for ventilation/perfusion single-photon emission computed tomography (SPECT) for diagnosis of pulmonary embolism and beyond. Eur J Nucl Med Mol Imaging.

[4] Skarlovnik A, Hrastnik D, Fettich J, Grmek M (2014). Lung scintigraphy in the diagnosis of pulmonary embolism: current methods and interpretation criteria in clinical practice. Radiol Oncol.

[5] Konstantinides SV, Meyer G, Becattini C, Bueno H, Geersing GJ, Harjola VP (2020). 2019 ESC guidelines for the diagnosis and management of acute pulmonary embolism developed in collaboration with the European Respiratory Society (ERS). Eur Heart J.

[6] Rahmanipour E, Ghorbani M, Sadeghi R, Sadraei N, Borhani A, Mohammadi S (2023). Diagnostic importance of lung perfusion/ ventilation scans in the evaluation of pulmonary embolism in COVID-19 patients: systematic review of the literature. Nucl Med Commun.

[7] Zuckier LS, Moadel RM, Haramati LB, Freeman LM (2020). Diagnostic evaluation of pulmonary embolism during the COVID-19 pandemic. J Nucl Med.

[8] Zuckier LS (2021). To everything there is a season: taxonomy of approaches to the performance of lung scintigraphy in the era of COVID-19. Eur J Nucl Med Mol Imaging.

[9] Lu Y, Lorenzoni A, Fox JJ, Rademaker J, Vander Els N, Grewal RK (2014). Noncontrast perfusion single-photon emission CT/CT scanning: a new test for the expedited, high-accuracy diagnosis of acute pulmonary embolism. Chest.

[10] Lu Y, Macapinlac HA (2020). Perfusion SPECT/CT to diagnose pulmonary embolism during COVID-19 pandemic. Eur J Nucl Med Mol Imaging.

[11] Le Roux PY, Le Gal G, Salaun PY (2020). Lung scintigraphy for pulmonary embolism diagnosis during the COVID-19 pandemic: does the benefit-risk ratio really justify omitting the ventilation study?. Eur J Nucl Med Mol Imaging.

[12] Schaefer WM, Knollmann D, Meyer PT (2021). V/Q SPECT/CT in the time of COVID-19: changing the order to improve safety without sacrificing accuracy. J Nucl Med.

[13] Lavely WC, Patel VK (2022). Ventilation-perfusion scans after the COVID-19 pandemic: counterpoint-ventilation studies are here to stay. AJR Am J Roentgenol.

[14] Vöö S, Dizdarevic S (2020). Single photon emission computed tomography lung perfusion imaging during the COVID-19 pandemic: does nuclear medicine need to reconsider its guidelines?. Nucl Med Commun.

[15] Karamian F, Sadeghi R, Askari E (2023). Lung ventilation-perfusion scan in COVID-19: various patterns of perfusion defects. Clinical Nuclear Medicine.

[16] Ghorbani M, Esmaeilian S, Rahmanipour E, Sadeghi R, Zahergivar A, Nikkhah R (2024). Diagnostic importance of performing myocardial perfusion imaging in COVID-19 patients: A systematic review. Iran J Nucl Med.

[17] Le Roux P-Y, Le Gal G, Salaun P-Y (2020). Lung scintigraphy for pulmonary embolism diagnosis during the COVID-19 pandemic: does the benefit-risk ratio really justify omitting the ventilation study?. Eur J Nucl Med Mol Imaging.

[18] Schaefer WM, Knollmann D, Meyer PT (2021). Q/V-SPECT CT in times of COVID-19: Changing the order to improve safety without sacrificing accuracy. J Nucl Med.

[19] Zuckier LS (2022). Safe Pulmonary Scintigraphy in the Era of COVID-19. Semin Nucl Med.

[20] Kumar A, Moadel RM, Haramati LB, Ye K, Freeman LM, Zuckier LS (2022). Experience with a perfusion-only screening protocol for evaluation of pulmonary embolism during the COVID-19 pandemic surge. J Nucl Med.

[21] Suh M (2022). In the COVID-19 era, is it ok to perform a perfusion-only SPECT/CT for the diagnosis of pulmonary embolism?. Nucl Med Mol Imaging.

[22] Pierre-Yves Le R, Pierre-Benoit B, Achraf B, Benoit D, Bertrand B, Caroline M-T (2022). Lung scintigraphy for pulmonary embolism diagnosis in COVID-19 patients: A multicenter study. J Nucl Med.

